# Anti-Angiogenic Treatments Interact with Steroid Secretion in Inflammatory Breast Cancer Triple Negative Cell Lines

**DOI:** 10.3390/cancers13153668

**Published:** 2021-07-21

**Authors:** Ángela Alonso-Diez, Sara Cáceres, Laura Peña, Belén Crespo, Juan Carlos Illera

**Affiliations:** 1 Department Animal Medicine, Surgery and Pathology, Veterinary Medicine School, Complutense University of Madrid (UCM), 28040 Madrid, Spain; angalo02@ucm.es (Á.A.-D.); laurape@vet.ucm.es (L.P.); 2 Department Animal Physiology, Veterinary Medicine School, Complutense University of Madrid (UCM), 28040 Madrid, Spain; belencre@ucm.es (B.C.); jcillera@ucm.es (J.C.I.)

**Keywords:** anti-angiogenic therapies, inflammatory breast cancer, IMC, IBC, steroid hormones

## Abstract

**Simple Summary:**

Inflammatory breast cancer (IBC) is the most aggressive breast cancer and is associated with poor prognosis. Exacerbated angiogenesis, lymphangiogenesis and lymphangiotropism are hallmarks of this tumour. Current antiangiogenic therapies have minimal effects on overall survival in IBC patients. Furthermore, it is well established that steroid hormones are strongly related to tumour development and progression, angiogenesis regulation and metastasis. We investigated the effect of different antiangiogenic therapies on steroid and angiogenic growth factor production using two inflammatory breast cancer cell lines. We reported that sex steroid hormones could regulate the production of angiogenic factors, since after the results, P4 and E2 were involved in VEGF production and androgens in the formation of vascular-like structures. Moreover, we reported that elevated intratumoural concentrations of T and E1SO4 could be associated with decreased metastatic rates and the promotion of tumour progression, respectively, and thus the measurement of sex steroids and growth factors may be useful to develop preventive and individualised therapeutic strategies.

**Abstract:**

Human inflammatory breast cancer (IBC) is a highly angiogenic disease for which antiangiogenic therapy has demonstrated only a modest response, and the reason for this remains unknown. Thus, the purpose of this study was to determine the influence of different antiangiogenic therapies on in vitro and in vivo steroid hormone and angiogenic growth factor production using canine and human inflammatory breast carcinoma cell lines as well as the possible involvement of sex steroid hormones in angiogenesis. IPC-366 and SUM149 cell lines and xenotransplanted mice were treated with different concentrations of VEGF, SU5416, bevacizumab and celecoxib. Steroid hormone (progesterone, dehydroepiandrostenedione, androstenedione, testosterone, dihydrotestosterone, estrone sulphate and 17β-oestradiol), angiogenic growth factors (VEGF-A, VEGF-C and VEGF-D) and IL-8 determinations in culture media, tumour homogenate and serum samples were assayed by EIA. In vitro, progesterone- and 17β-oestradiol-induced VEGF production promoting cell proliferation and androgens are involved in the formation of vascular-like structures. In vivo, intratumoural testosterone concentrations were augmented and possibly associated with decreased metastatic rates, whereas elevated E1SO4 concentrations could promote tumour progression after antiangiogenic therapies. In conclusion, sex steroid hormones could regulate the production of angiogenic factors. The intratumoural measurement of sex steroids and growth factors may be useful to develop preventive and individualized therapeutic strategies.

## 1. Introduction

Normal and neoplastic mammary glands are considered endocrine tissues due to the local biosynthesis of steroid hormones [1,2,3,4]. Oestrogens are essential for breast development and maintenance by binding to receptors: oestrogen receptor alpha (ERα) and oestrogen receptor beta (ERβ) [5]. Several studies have demonstrated a strong association between elevated levels of circulating oestrogens and their metabolites with an increased risk of breast cancer development [6,7,8,9,10]. Moreover, experimental data support that oestrogen signalling by ERα in breast cancer results in DNA damage, cellular proliferation and decreased apoptosis [11,12].

On the other hand, the role of androgens and their receptors in breast cancer development and progression is a debated topic. Prospective epidemiologic studies have shown that circulating androgens in postmenopausal women are positively associated with breast cancer, based on their role as oestrogenic precursors [13,14]. However, data from in vitro and in vivo studies suggest that they may also exert an antiproliferative and apoptotic effect [13,15].

In order to grow and metastasise, tumours require an adequate blood supply of oxygen and nutrients. Thus, angiogenesis, the formation of new blood vessels from pre-existing ones, is critical in the development, progression, and metastasis of tumours [16,17]. The angiogenic process (sprouting angiogenesis) is relatively complex and characterised by an angiogenic switch in the dynamic balance between proangiogenic and antiangiogenic factors, shifted towards an irreversible proangiogenic state, leading to the recruitment of a new vascular supply [17]. Among the proangiogenic factors, the vascular endothelial growth factor family (VEGF) and their receptors (VEGFRs) stand out [18] playing critical roles in initiating and promoting angiogenesis [19].

However, apart from these angiogenic factors, data on the effects of sex steroid hormones on this angiogenic switch is emerging in the literature. Although a clear picture is not still available, several recent investigations suggest that both oestrogen and progesterone are involved in the process of angiogenesis by their regulatory effects on VEGF and its receptors [20,21].

Since angiogenesis has become an important target, there has been intensive research in order to determine the proteins or mediators involved in prompting angiogenesis and finding effective antiangiogenic drugs that can decrease the release of pro-angiogenic factors, prevent their binding to receptors or inhibit their actions [17,22]. Specifically, antiangiogenic therapies against the VEGF family block either the ligands or the receptors [23]. The most widely studied antiangiogenic therapeutic is bevacizumab, a humanised monoclonal antibody that binds with high affinity to VEGF-A preventing its binding to its receptor, thus inhibiting tumour vascular endothelial cell proliferation and angiogenesis, reducing vascular permeability, and promoting tumour blood vessel degradation [24]. Small-molecule tyrosine kinase inhibitors (TKIs) that block the VEGF family receptors have also been developed. For instance, SU5416 is a small molecule inhibitor of VEGFR-2 and other several tyrosine kinase receptors (TKRs), including VEGFR-1, c-Kit and Flt-3 [25], that exerts an antiproliferative effect on cultured endothelial cells, and inhibits tumour growth and decreases vascular density in xenograft models [26,27].

Moreover, over the past two decades, researchers have identified some molecular changes that also play important roles in breast cancer, such as COX-2, becoming a potential chemoprevention target for breast cancer. Therefore, selective COX-2 inhibitors, such as celecoxib, emerged as promising anticancer drugs in the treatment and prevention of breast cancer due their multiple potential antitumour mechanisms, including the inhibition of proliferation, induction of apoptosis and antiangiogenic effects among others [28,29,30,31,32,33,34,35,36].

Inflammatory breast cancer (IBC) is the most lethal and aggressive spontaneous form of locally advanced breast cancer with a high rate of metastases [35,37] and is characteristically highly angiogenic and angioinvasive [38]. Furthermore, IBC is associated with abnormal mRNA VEGF levels, high circulating VEGF and stromal VEGF expression [35,39], and it has been established that high COX-2 expression correlates with worse overall survival (OS) and higher nuclear grade in IBC patients [35]. For this special type of breast cancer, these current antiangiogenic therapies have minimal effects on overall survival in IBC patients, and the reason for this remains unknown.

Several studies have revealed that canine inflammatory mammary cancer (IMC) is a good spontaneous animal model for the study of IBC [3,40,41]. IPC-366, a uniquely established canine IMC cell line [42], has demonstrated to be a good model in comparison with its human counterpart SUM149 [15]. Exacerbated angiogenesis, lymphangiogenesis, lymphangiotropism and vasculogenic mimicry (VM) have been similarly observed in both cell lines [42], so they represent interesting models for the study of angiogenesis in this special type of tumour [43].

Since antiangiogenic therapies have shown limited results and the production of steroid hormones is strongly related to tumour development, progression and angiogenesis regulation, the aim of the present study was to determine the influence of different anti-angiogenic therapies on in vitro and in vivo steroid production using the IBC and IMC cancer cell lines to improve current knowledge of this type of breast tumour and the possible influence of sex steroid hormones on the modest responses of these therapies.

## 2. Materials and Methods

### 2.1. Cell Line Culture

The triple-negative canine IMC cell line, IPC-366, was obtained from the Department of Animal Physiology of Veterinary Medicine School of the Complutense University of Madrid, Spain, and was cultured in Dulbecco’s modified Eagle medium nutrient mixture F-12 Ham (DMEM/F12; Sigma Aldrich, D6421) supplemented with 5% fetal bovine serum (FBS; Sigma Aldrich, St. Louis, MO, USA, F7524), 1% penicillin–streptomycin solution (Sigma Aldrich, P0781) and 1% L-glutamine (Sigma Aldrich, G7513).

The SUM149 triple-negative human IBC cell line was originally obtained from Asterand, Plc. (Detroit, MI, USA) and was grown in Ham’s F-12 medium (Invitrogen, 21765029) supplemented with 5% FBS (Sigma Aldrich, 12103C), 1 ug/mL hydrocortisone (Sigma Aldrich, H4001), 5 ug/mL insulin (Sigma Aldrich, I9278) and 1% penicillin-streptomycin solution (Sigma Aldrich, P0781).

Both cell lines were cultured in 25 cm^2^ culture flasks and maintained in a humidified 5% carbon dioxide (CO_2_) atmosphere at 37 °C. The cell cultures were observed daily by phase-contrast microscopy (Optika XDS-2 Inverted Microscope, Euromicroscopes, S.L, Barcelona, Spain) to check cell viability and growth.

### 2.2. In Vitro Treatments

VEGF (specifically, VEGF165 isoform, which shares amino acid sequences between human and canine species [44]), a key mediator of angiogenesis; the RTK inhibitor SU5416; and the selective COX-2 inhibitor, celecoxib, were obtained from Sigma Aldrich (Madrid, Spain). The anti-VEGF drug, bevacizumab, was kindly supported by Genentech Inc. (San Francisco, USA).

All drugs were dissolved in dimethyl sulfoxide (DMSO), stored at −20 °C and diluted in the corresponding fresh culture medium immediately before use.

Cultured IPC-366 and SUM149 cells were divided into a control group, treated with DMSO (final concentration, <0.1%), and four experimental groups, in which different concentrations of the different drugs tested were added to the culture medium.

To determine the sensitivity of IPC-366 and SUM149 cells to the effects of VEGF, SU5416, bevacizumab and celecoxib, different concentrations were used to determine the critical final concentrations to be used in the MTS assay.

For in vitro treatments, IPC-366 and SUM149 cells were exposed to 0.62, 1.25 and 2.5 uM concentrations of VEGF. SU5416, bevacizumab and celecoxib at 1.5, 3 and 6 uM final concentrations were added to the culture medium.

### 2.3. Cell Viability Assay (MTS Assay)

To evaluate cell viability, IPC-366 and SUM149 were assayed using the CellTiter 96 Aqueous One Solution Cell Proliferation Assay, according to manufacturer’s instructions (Promega, Madrid, Spain). Briefly, a total of 1 × 10^6^ IPC-366 and SUM149 cells per well were seeded in 96-well plates, and the different concentrations of all studied drugs were added. After 24, 48 and 72 h, the media were removed, and 100 uL of media containing 20 uL of MTS was added to each well and incubated at 37 °C for 3 h in a humidified, 5% CO_2_ atmosphere. Absorbance was recorded at 490 nm using a 96-well SpectraMax 190UV/Vis plate reader. Untreated cells were considered to represent 100% proliferation, and all drug-treated cells were expressed relative to this. Each experiment was performed in quadruplicate and repeated at least three times.

The culture supernatants of untreated and treated cells removed at 24, 48 and 72 h were collected and frozen at −20 °C until hormone analysis.

### 2.4. Tube Formation Assay

Tube formation assays were performed as described by Guo et al. [45]. Briefly, growth factor-reduced Matrigel (Sigma Aldrich, E6909) was plated on the bottom of a 24-well plate (Cornings Costars TC-treated Multiple Well Plates, Ref 353047) and left at 37 °C for 30 min for solidification. Thereafter, a total of 5 × 10^4^ IPC-366 and SUM149 cells were seeded on previously coated matrigel wells with their corresponding culture media containing 0.25% BSA. Both IPC-366 and SUM149 cells were divided into a control group and 4 experimental groups, treated with a final concentration of 1.5 uM for VEGF, SU5416, bevacizumab and celecoxib, respectively. Plates were incubated at 37 °C in 5% CO_2_ conditions for 12 h. Optical images of the wells were taken at 6 h at 10× magnification with a phase-contrast microscopy (Optika XDS-2 Inverted Microscope, Euromicroscopes, S.L, Barcelona, Spain). Incomplete networks were excluded, and vessel-like structures were quantified by counting vessel-like tubes in each well using the ImageJ Angiogenesis Analyzer software. The experiments were repeated two times, each time with duplicate wells. At the end of the assay, the corresponding culture media of untreated and treated cells of both cell lines were collected and stored at −20 °C until hormone analysis.

### 2.5. Experimental Animals and Treatments

In total, 130 female Balb/SCID mice were obtained from Harlem Laboratories Models, SL (Barcelona, Spain), early in the morning with dams to minimize shipping stress and adapted for 7 days in the Animal Facility (Animal Physiology Department, Veterinary Medicine School, Complutense University of Madrid (UCM)). The mice were housed in polycarbonate cages (one to two animals per cage) in a room with controlled environmental conditions (temperature: 23 ± 2 °C; relative humidity: 50 ± 10%; 10–15 air changes per hour; and 12:12 h light:dark cycle). Soy-free pellet food (Dyets, Inc, Bethlehem, Pennsylvania) and water, previously sterilised, were provided ab libitum. The required sample size needed to simultaneously compare the normal means of the five experimental groups was determined using the sample size determination module of the statistical package Statgraphics Centurion XVI (Statpoint Technologies, Inc., Warrenton, Virginia) resulting in a total of five mice per group studied. Experimental protocols of this study were approved by the Institutional Animal Care and Use Committee of the Complutense University of Madrid, Spain (number: Proex 31/15). All procedures were completed in accordance with the Guide for the Care and Use of Laboratory Animals and conformed to the relevant EU Directive.

A suspension of 10^6^ IPC-366 and SUM149 cells in 200 uL of phosphate-buffered saline (PBS) was subcutaneously inoculated in the left ventral region of 6-8-week-old female Balb/SCID mice with a 21-gauge syringe. Mice were inspected twice weekly for the development of the tumours until a volume of 0.5 cm^3^ was reached. Once the previous volume was reached, mice were divided into five experimental groups: a control group (*n* = 10 mice, five mice of IPC-366 and five mice of SUM149), injected with DMSO, and four experimental groups (*n* = 15 mice per group, five mice per dosage), injected with the corresponding dosage of each treatment. The dosages employed per treatment were established as low, medium and high for VEGF (0.2, 0.4 and 1 mg/kg), SU5416 and bevacizumab (1, 5 and 10 mg/kg) and celecoxib (0.5, 2.5 and 5 mg/kg), respectively, and were chosen based on the literature [46,47,48,49].

For treatment administration, mice were intraperitoneally injected in the right ventral region with a 21-gauge syringe every three days for a total of 15 days. The tumour volumes were assessed every three days by measuring the length and width with callipers and estimated using the following formula: volume = (width)/2 × (length)/2, where the width is the smaller of the two dimensions [50].

When tumours reached a volume of 1.5 cm^3^ (end-point) or at the end of treatment, blood samples were obtained intracardially using a 1 mL syringe with a 25-gauge needle and collected in heparin-coated tubes (S-Monovette^®^ Lithium-Heparin Gel+, Sarstedt, Spain). Prior to this procedure, animals were anaesthetised with isoflurane (IsoVet) at 4% for induction and 1.5% for maintaining sedation, supplied at a fresh gas flow rate of 0.5 L of oxygen/minute. After blood collection, animals were euthanised by a lethal dose of isoflurane and tumours and organs were harvested at necropsy for subsequent analysis.

### 2.6. Histological Examination

To assess tumour invasion to distant organs and metastatic ability, lungs and livers were collected and fixed in 10% buffered formalin solution (pH 7.4) for 24 h. Then, samples were trimmed, embedded in paraffin wax, sectioned at 3 µM thickness and stained with haematoxylin and eosin (H&E) for light microscopic examination. As no visible metastatic nodules were found, micrometastases were scored in haematoxylin and eosin (H&E)-stained sections of paraffin-embedded tissues. Specifically, the micrometastases were verified at ×10 magnification and counted at ×40 magnification. Mean scores of lungs and liver micrometastasis were based on five mice per group, with each score acquired from counts of all pulmonary and hepatic lobes. As no specific marker was used for tumour cells, metastasis at the single cell level or as small cell clusters could not be accurately determined, unless cancer cells appeared as overt colonies. Both pulmonary and hepatic metastases were scored by two independent trained pathologists (A.A.D and L.P).

### 2.7. Steroid Determinations in Culture Media, Serum and Tumour Homogenates

For tumour homogenates, a total of 0.5 gr of tumour collected at necropsy was homogenised in phosphate buffered saline (PBS) (pH 7.2), and centrifugated at 1200× *g*, for 20 min at 4 °C. Supernatants were collected, aliquoted individually and frozen at −80 °C until hormones were assayed. Blood samples were centrifugated at 1200× *g* and 4 °C for 20 min, and the serum was separated and stored frozen at −20 °C until assayed.

The hormones evaluated and antibodies used are summarised in Table 1. The assayed steroid hormones were progesterone (P4), dehydroepiandrosterone (DHEA), androstenedione (A4), testosterone (T), dihydrotestosterone (DHT), oestrone sulphate (E1SO4) and 17beta-oestradiol (E2). Moreover, the angiogenic factors determined were VEGF-A, VEGF-C, VEGF-D and IL-8. Steroid hormone determinations (P4, A4, T, E1SO4 and E2) in tumour homogenates were assayed by previously validated competitive enzyme-immunoassay (EIA) [51], whereas these determinations in culture media and serum samples were assayed by a competitive amplified EIA previously validated in our laboratory [43]. Briefly, 96-well flat-bottom medium binding polystyrene microplates (Biohit, Finland) were coated overnight at 4° C with the appropriate purified antibody dilutions. Afterwards, plates were washed, and conjugate working solutions (CWS) were prepared. For competitive EIA, standards and tumour homogenate samples were diluted in CWS, analysed in duplicate and incubated at room temperature for 2 h to achieve a competitive reaction. For amplified EIA, standards as well as culture and serum samples were added in duplicate to the plate and incubated overnight at 4° C. After that, CWS was added to each well and incubated for 4 more hours at room temperature. For both EIAs, after conjugate incubation plates were washed, and to evaluate the amount of labelled steroid hormones, Enhance K-Blue TMB substrate (Neogen, Lexington, KY, USA) was added to each well and incubated for an additional 15 min at room temperature. Finally, the colorimetric reaction was stopped by the addition of 10% H_2_SO_4_ to each well. Absorbance was read at 450 nm using a 96-well SpectraMax 190UV/Vis automatic plate reader. Hormone concentrations were calculated by means of software developed for this technique (ELISA AID, Eurogenetics, Belgium). A standard dose–response curve was constructed by plotting the binding percent (B/B0 × 100) against each steroid hormone standard concentrations. The validation technique parameters: percentage of cross-reactivity of polyclonal antibodies against related steroids, recovery rates, sensitivity, intra- and interassay coefficients of variation and parallelism were assayed as previously reported by Illera et al. [43]. P4, A4, T, E1SO4 and E2 antibodies with specificity against human and canine steroids were developed by the Department of Animal Physiology, Veterinary Medicine School, Complutense University of Madrid (UCM), Spain. On the other hand, DHT, DHEA, VEGF-A, VEGF-C, VEGF-D and IL-8 determinations were performed using a commercially available EIA kit with confirmed cross reactivity to canine and human steroids and proteins, according to the manufacturer’s recommendations (Table 1).

All hormone concentrations were expressed in ng/g for tumour homogenates, and ng/mL in the case of serum samples and culture media, except for DHT culture media hormone concentrations, which were expressed in pg/mL.

### 2.8. Statistics

The Kolmogorov–Smirnoff test was used to assess the goodness-of-fit distribution of the hormonal data, growth factors and foci of metastasis. Since hormones and growth factor data were noted to be non-parametric, the Kruskal–Wallis test was used to compare data. For comparison between control and treatment group results of both cell lines, we used a non-parametric test Wilcoxon’s rank sum test with SAS 9.4. Data are shown as the mean ± standard error (SE). For tumour growth, differences between the experimental group means were analysed by one-way analysis of variance (ANOVA). Foci of metastasis among control and treated groups were compared through an Unpaired Student’s *t*-test when the distribution of data was normal, and by the Mann–Whitney U test when the distribution was non-parametric. In all statistical comparisons, *p* values < 0.05 were considered statistically significant.

## 3. Results

### 3.1. Anti-Proliferative Effect on In Vitro Cell Viability with the Addition of Anti-Angiogenic Treatments

The effects of the addition of the antiangiogenic treatments into the culture medium, demonstrated that VEGF and SU5416 promote increased cell viability; however, celecoxib and bevacizumab decrease it.

**Figure 1:**
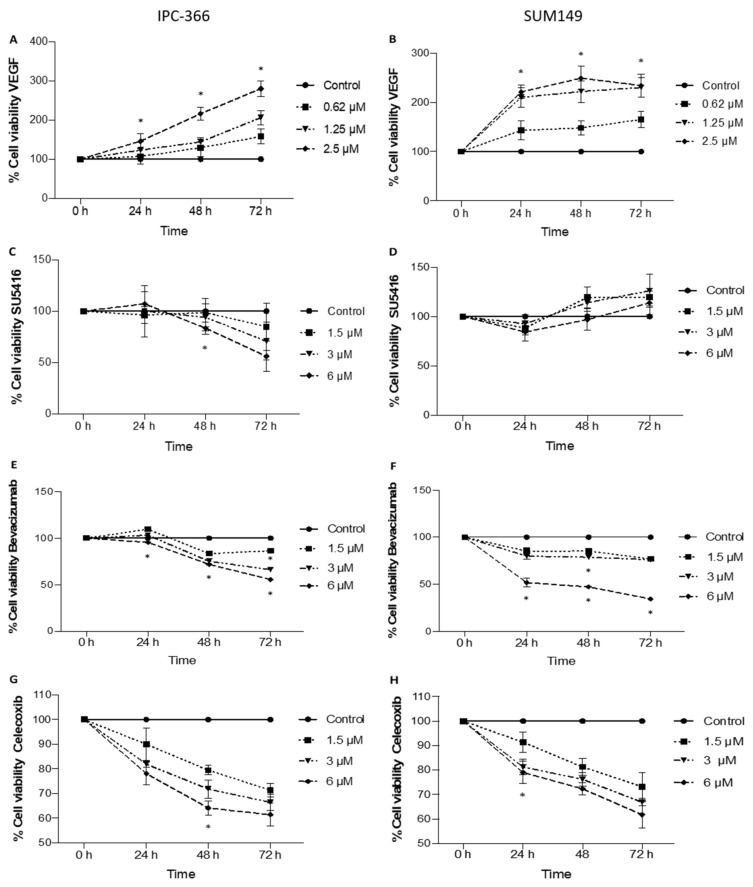
In vitro VEGF (0.62, 1.25, 2.5 uM), SU5416, bevacizumab and celecoxib (1.5, 3, 6 uM) effects on cell viability after 24 h, 48 h and 72 h post-treatment in IPC-366 and SUM149 cell lines measured by MTS assay. Cell viability increases after VEGF addition in both cell lines (**A**,**B**). Cell viability decreased after SU5416 treatment in IPC-366 cells and increased in SUM149-treated cells (**C**,**D**). Decreased cell viability in IPC-366- and SUM149 treated cells after bevacizumab (**E**,**F**) and celecoxib (**G**,**H**) addition. Data are shown as means ± SEM. * Significant differences between control and treated groups (*p* < 0.05).

Specifically, the addition of VEGF to the culture medium of IPC-366 and SUM149 significantly augmented (*p* < 0.05) cell viability in all studied dosages, increasing with the dose and time post-treatment in IPC-366; however, in SUM149, after 24 h, cell viability was maintained (Figure 1A,B).

On the other hand, as in IPC-366 cells there was a reduction in the viability percentage in all studied dosages after SU5416 addition; in SUM-149, the percentage of viable cells increased, although these differences were not statistically significant (Figure 1C,D). Regarding bevacizumab and celecoxib results in both cell lines, the percentage of viability diminished in all studied dosages, with the most reduction observed for the highest dose (Figure 1E–H).

### 3.2. Anti-Angiogenic Treatments Alter In Vitro Steroid Hormone Secretion

**Figure 2:**
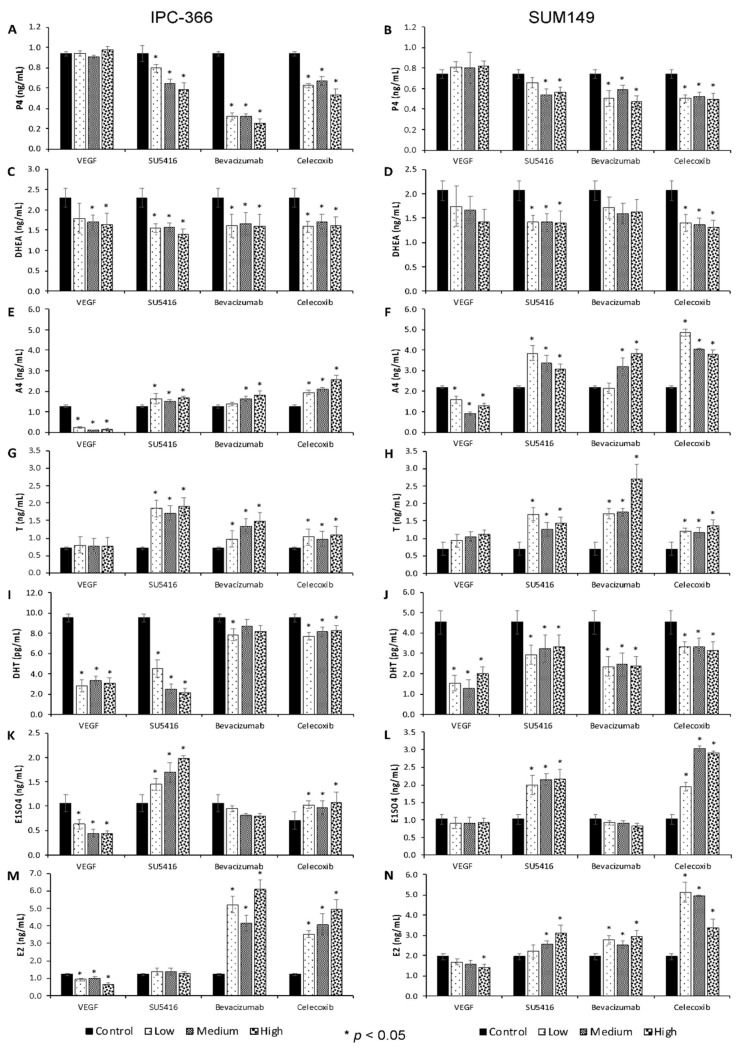
Sex steroid hormone concentrations in culture media of control and treated IPC-366 and SUM-149 cells at 72 h after the addition of low, medium, and high concentrations of VEGF (0.62 uM, 1.25 uM and 2.5 uM); SU5416, bevacizumab and celecoxib (1.5 uM, 3 uM and 6 uM). (**A**,**B**) Progesterone (P4). (**C**,**D**) Dehydroepiandrosterone (DHEA). (**E**,**F**) Androstenedione (A4). (**G**,**H**) Testosterone (T). (**I**,**J**) Dihydrotestosterone (DHT). (**K**,**L**) Estrone sulphate (E1SO4). (**M**,**N**) Estradiol (E2). Bar represents means ± SEM. * Significant differences between control and treatment groups (*p* < 0.05).

The steroid hormone (P4, DHEA, A4, T, DHT, E1SO4 and E2) concentrations of IPC-366 and SUM149 were measured in the cultured media of treated and untreated cells (Figure 2; Table S1).

Similar secretion patterns were found among both cell lines. Results showed that the addition of antiangiogenic treatments (SU5416, bevacizumab and celecoxib) decreased P4 secreted levels with respect to the control group.

Regarding the steroid precursors (DHEA and A4) and androgens (T and DHT), the results demonstrated that DHEA and DHT levels were significantly decreased (*p* < 0.05) in all treatments in both cell lines. With regard to A4 and T concentrations, a significant increase (*p* < 0.05) was found after SU5416, bevacizumab and celecoxib treatments; meanwhile, VEGF addition provoked a decrease in only A4 levels.

On the other hand, the levels of both steroid oestrogens, E1SO4 and E2, were significantly augmented (*p* < 0.05) in SU5416 and celecoxib; however, after bevacizumab and celecoxib addition, only E2 concentrations were significantly increased (*p* < 0.05).

**Figure 3:**
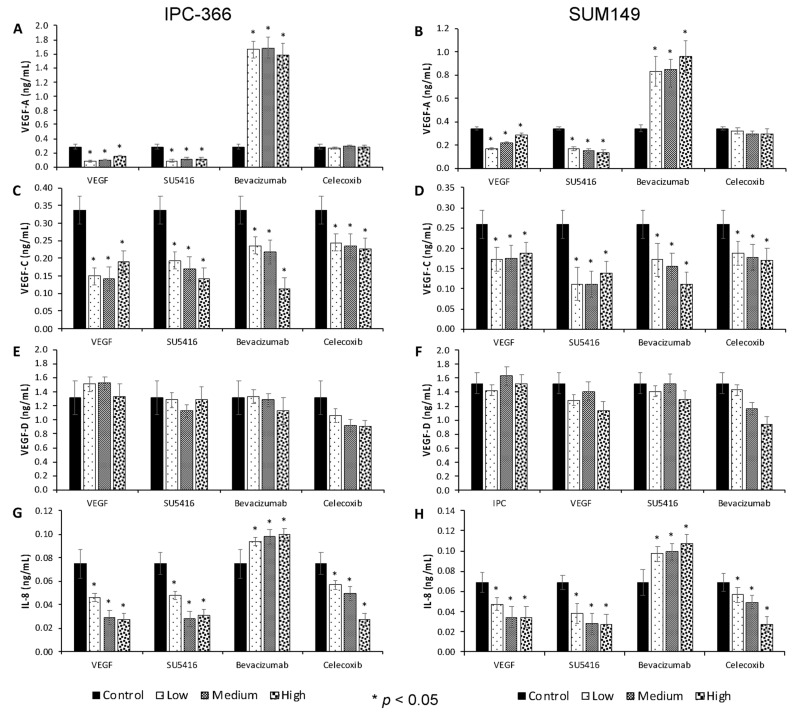
Angiogenic growth factors and interleukin IL-8 concentrations in culture media of control and treated IPC-366 and SUM-149 cells at 72 h after the addition of low, medium and high concentrations of VEGF (0.62 uM; 1.25 uM and 2.5 uM); SU5416, bevacizumab and celecoxib (1.5 uM, 3 uM, and 6 uM). (**A**,**B**) Vascular endothelial growth factor A (VEGF-A). (**C**,**D**) Vascular endothelial growth factor C (VEGF-C). (**E**,**F**) Vascular endothelial growth factor D (VEGF-D). (**G**,**H**) Interleukin-8 (IL-8). Bar represents means ± SEM. * Significant differences between control and treatment groups (*p* < 0.05).

The addition of the antiangiogenic treatments and VEGF also altered the secretion of the angiogenic growth factors and IL-8 (Figure 3, Table S1). Specifically, our results showed a significant decrease (*p* < 0.05) in VEGF-A levels in cells treated with VEGF and SU5416; however, these concentrations were significantly augmented (*p* < 0.05) in cells treated with bevacizumab. With regard to VEGF-C, the concentrations were decreased in all treatments. On the other hand, while the addition of VEGF, SU5416 and bevacizumab did not exert any effect on VEGF-D levels, these levels were significantly decreased (*p* < 0.05) with celecoxib. Finally, IL-8 levels were decreased (*p* < 0.05) in VEGF, SU5416 and celecoxib-treated cells, whereas with bevacizumab, the concentrations significantly increased (*p* < 0.05).

### 3.3. Effect of Anti-Angiogenic Compounds and VEGF in Tube Formation

**Figure 4:**
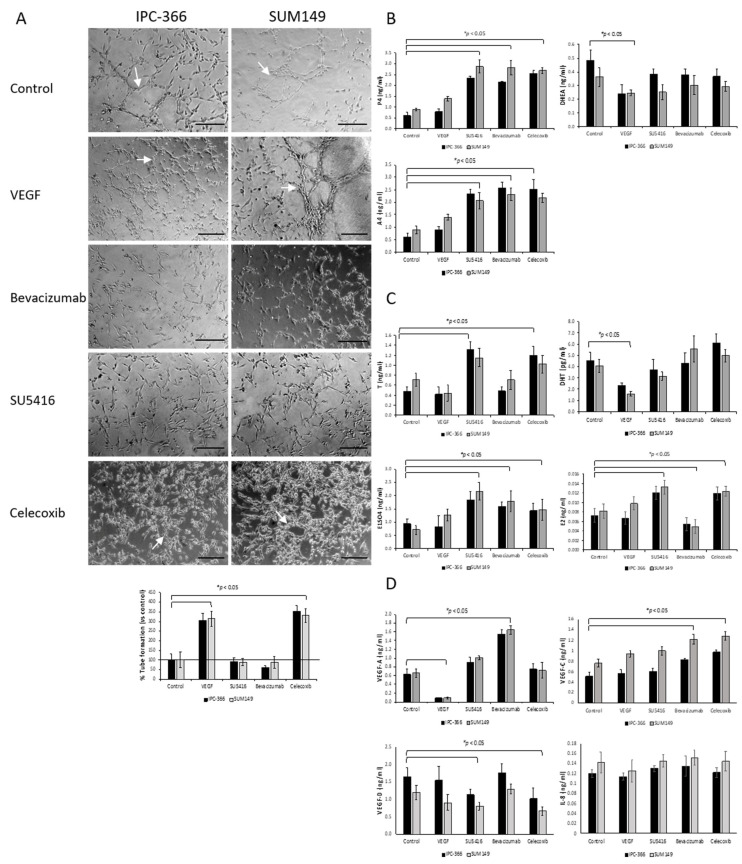
Tube formation results. (**A**) Images ×10 of IPC-366 and SUM149 vascular-like structures (arrows) formed in control and treated groups treated with a final concentration of 1.5 uM for VEGF, SU5416, bevacizumab and celecoxib. Bar graph represents percentage of number of tubes formed at 6 h respect to control group. (**B**) Steroid precursor (P4, DHEA, A4); (**C**) sexual steroids (T, DHT, E2, E1SO4) and (**D**) angiogenic growth factors (VEGF-A, VEGF-C, VEGF-D, IL-8) in culture media of IPC-366 (black) and SUM149 (grey) after tube formation assay. * Denoted significant differences (*p* < 0.05) between control and treated groups. Scale bar 200 µM.

To further characterize the role of the different antiangiogenic treatments, we focused on another phenotypic characteristic of angiogenesis in vitro, namely, the formation of vascular-like structures by neoplastic cells by conducting a tube formation assay. As shown in Figure 4, VEGF and celecoxib significantly increased (*p* < 0.05) the number of tubular-like structures with respect to the control group. However, treatment with bevacizumab and SU5416 resulted in a slight decrease in tube formation.

After the tube formation assay, steroid hormone concentrations (P4, DHEA, A4, T, DHT, E1SO4 and E2), angiogenic growth factors (VEGF-A, VEGF-C and VEGF-D) and IL-8 concentrations were also evaluated to determine the possible impact on the cell tube formation (Figure 4).

Steroid precursor P4 concentrations were significantly higher (*p* < 0.05) in cells treated with SU5416, bevacizumab and celecoxib with respect to the control group. However, a significant reduction in DHEA and DHT secreted levels (*p* < 0.05) was found after VEGF addition. Regarding A4 and T levels, results demonstrated significantly higher (*p* < 0.05) concentrations in SU5416- and celecoxib-treated cells with respect to the control group. Oestrogen levels (E1SO4 and E2) were significantly increased (*p* < 0.05) after SU5416 and celecoxib; however, after bevacizumab addition, E1SO4 levels increased and E2 levels decreased.

Regarding angiogenic growth factors and IL-8 secretion, the results revealed that VEGF significantly diminished (*p* < 0.05) VEGF-A concentrations. However, these VEGF-A levels were significantly higher (*p* < 0.05) after bevacizumab addition. Moreover, VEGF-C secreted concentrations were significantly augmented (*p* < 0.05) after bevacizumab and celecoxib; meanwhile, VEGF-D levels diminished (*p* < 0.05) after SU5416 and celecoxib treatment

### 3.4. VEGF, SU5416, Bevacizumab and Celecoxib Effects on Tumour Progression and Metastasis

**Figure 5:**
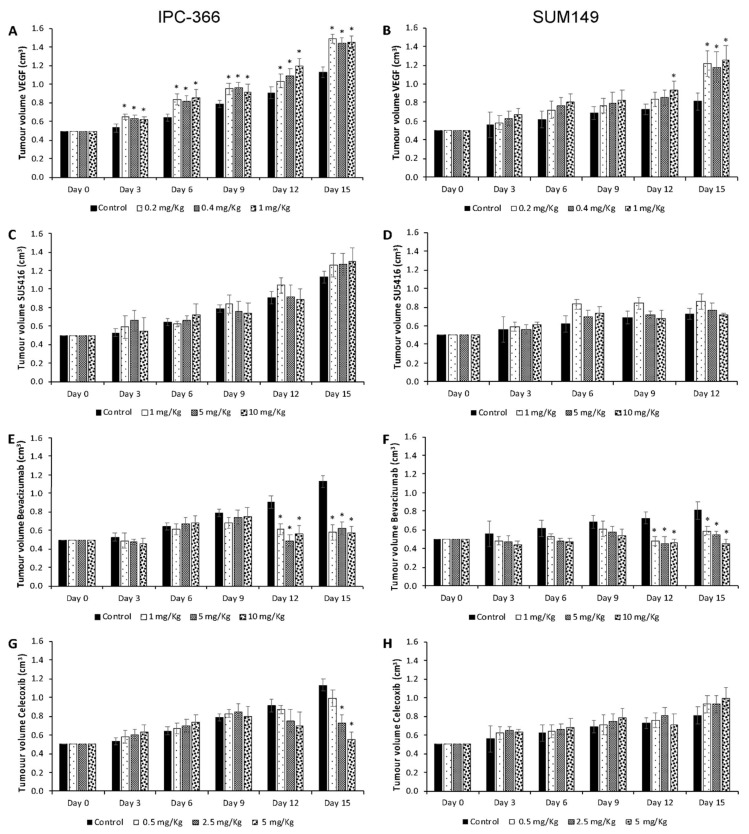
In vivo effect of VEGF, SU5416, bevacizumab and celecoxib on tumour growth in xenotransplanted mice of (**A**,**C**,**E**,**G**) IPC-366 and (**B**,**D**,**F**,**H**) SUM149. Bar represents means ± SEM. * Significant differences between control and treatment groups (*p* < 0.05).

**Figure 6:**
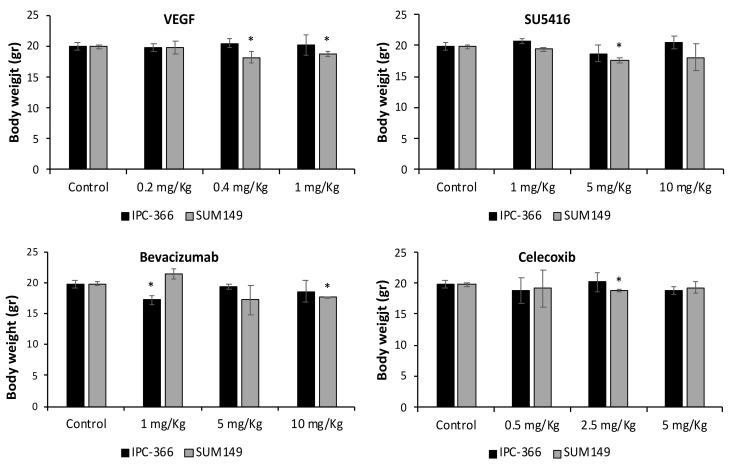
Body weight from xenotransplanted mice. Bar represents means ± SEM. * Significant differences between control and treatment groups (*p* < 0.05).

Figure 5 and Supplementary Figures S1 and S2 represent the effect of the different treatments on tumour growth in xenotransplanted mice with IPC-366 and SUM149 (Figure 6).

Comparing the effects of the different antiangiogenic therapies and VEGF in xenotransplanted mice, our results revealed that bevacizumab was the only treatment that significantly decreased (*p* < 0.05) tumour growth in IPC-366 and SUM149 xenografts. On the contrary, VEGF administration significantly increased (*p* < 0.05) tumour growth in both cell line-xenografted mice.

No significant differences were found after SU5416 and celecoxib treatment with respect to the control group, except in IPC-366 xenotransplanted mice treated with medium and high dosages of celecoxib, in which tumour progression decreased significantly (*p* < 0.05) from day 12.

**Figure 7:**
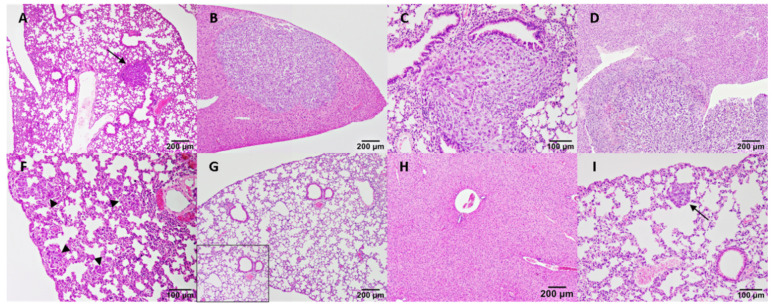
Metastatic foci in lungs and liver after VEGF, SU5416, bevacizumab and celecoxib treatment in IPC-366 and SUM149 xenotransplanted mice. (**A**) SUM149 control mice lung with metastatic focus. (**B**) IPC-336 control mice liver with large metastic focus. (**C**,**D**). Foci of metastases in lung and liver after VEGF inoculation in IPC-366- (c) and SUM149- (d) xenotransplanted mice. (**F**) Multiple small foci of metastasis in the lung of SUM-149 mice treated with 5 mg/kg of SU5416. (**G**,**H**). Absence of metastatic foci after bevacizumab treatment in IPC-366 xenografted mice. (**I**) Small foci of metastasis after inoculation (0.5 mg/kg) of celecoxib.

Regarding metastasis (Table 2, Figure 7), VEGF augmented the number of metastatic foci in the lungs and liver with respect to the control group in both xenotransplanted mice; these differences were only statistically significant for the high dosage.

After SU5416 inoculation, the number of metastatic foci found in lungs and liver was slightly lower for IPC-366-xenotransplanted mice but slightly higher in the case of SUM149 mice compared to the control group. Regarding bevacizumab and celecoxib, the number of metastatic foci in both organs was decreased for both treatments. Specifically, no pulmonary and hepatic metastatic foci were found for the high dosage of bevacizumab in IPC-366 xenotransplanted and medium and high dosages in SUM149 mice (Table 2). In the case of celecoxib, no hepatic foci of metastasis were found for medium and high dosages in IPC-366 mice and for any of the dosages in the case of SUM149-xenotransplanted mice.

### 3.5. Steroid Determinations in Tumour Homogenates and Serum Samples

**Figure 8:**
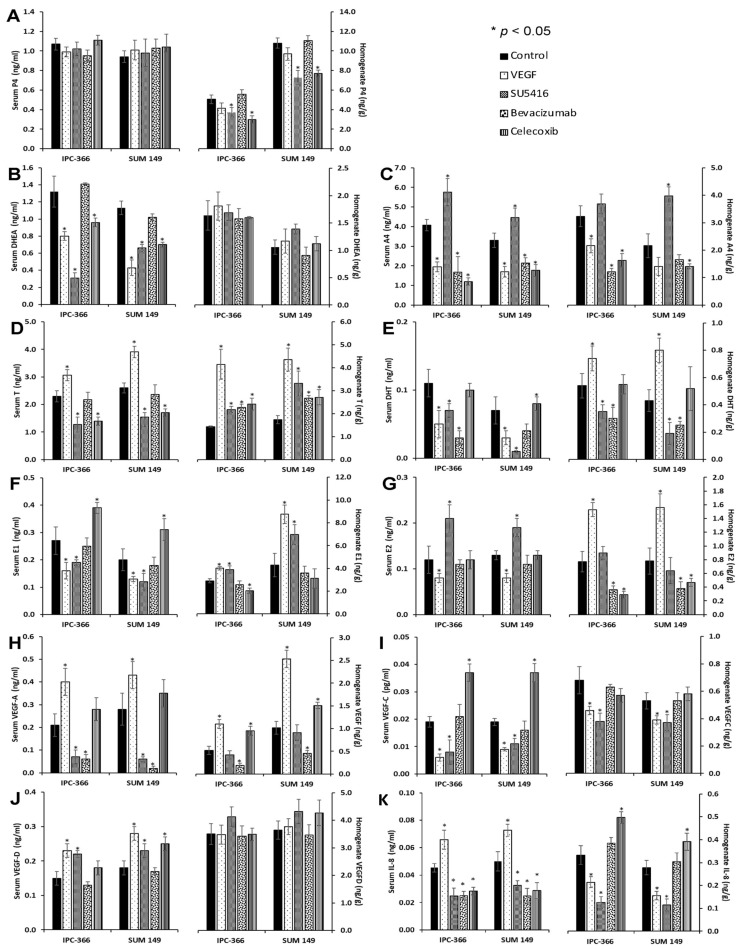
In vivo tumour homogenate and serum sex steroid hormones, angiogenic growth factors and IL-8 concentrations in IPC-366- and SUM149 xenotransplanted mice after inoculation of the high dosage of VEGF (1 mg/kg), SU5416 (10 mg/kg), bevacizumab (10 mg/kg) and celecoxib treatment (5 mg/kg). Bar represents means ± SEM. * Significant differences between control and treatment groups (*p* < 0.05). (**A**) Serum P4 levels were no altered but its intratumoral concentrations decreased after SU5416 and celecoxib. (**B**) Serum DHEA levels decreased after VEGF, SU5416 and celecoxib and no changes in its intratumoral concentrations were found after all treatments. (**C**) Serum and intratumoral A4 levels decreased after VEGF, bevacizumab and celecoxib. (**D**) Serum T levels decreased after SU5416 and celecoxib, but tumour homogenate levels were higher after all treatments. (**E**) Both serum and intratumoral DHT levels decreased after SU5416 and bevacizumab. (**F**) VEGF and SU5416 inoculation diminished circulating E1SO4 levels but augmented its intratumoral concentrations. (**G**) Serum E2 levels decreased after VEGF but augmented after SU5416, however, tumour homogenate concentrations were higher after VEGF but decreased after bevacizumab and celecoxib (**H**) Circulating and intratumoral VEGF-A levels augmented after VEGF inoculation, but these concentrations decreased only after bevacizumab. (**I**) VEGF and SU5416 treatments decreased circulating VEGF-C levels and celecoxib augmented them, however, only VEGF and SU5416 decreased intratumoral concentrations. (**J**) Only serum VEGF-D levels were augmented after VEGF and SU5416 treatments. (**K**) Serum IL-8 levels decreased after the inoculation of SU5416, bevacizumab and celecoxib, whereas its tumour levels decreased after VEGF and SU5416 but augmented after celecoxib.

The results from tumour homogenates and serum steroid concentrations of IPC-366 and SUM149 (Figure 8 and Table S2) revealed that both cell lines follow the same pattern.

Antiangiogenic treatments and VEGF inoculation did not alter P4 serum production. However, P4 tumour homogenate levels showed a significant decrease (*p* < 0.05) when both mice xenografts were treated with SU5416 and celecoxib. Regarding precursor steroids (DHEA and A4), the results showed that only serum DHEA concentrations were decreased (*p* < 0.05) after VEGF, SU5416 and celecoxib, whereas serum and tumour homogenate A4 concentrations significantly decreased (*p* < 0.05) after VEGF, bevacizumab and celecoxib. Serum T levels significantly decreased (*p* < 0.05) after SU5416 and celecoxib inoculation, but tumour homogenate concentrations were higher (*p* < 0.05) after all treatments. Regarding DHT levels, SU5416 and bevacizumab significantly decreased (*p* < 0.05) DHT serum and tumour homogenate concentrations. On the other hand, the results revealed that VEGF significantly diminished (*p* < 0.05) oestrogen circulating levels (E1SO4 and E2) and augmented tumour production; however, only celecoxib and bevacizumab significantly reduced (*p* < 0.05) E2 tumour homogenate concentrations.

Regarding VEGF-A levels, VEGF inoculation augmented (*p* < 0.05) serum and tumour homogenate VEGF-A concentrations, but only bevacizumab significantly reduced (*p* < 0.05) both serum and tumour levels. Moreover, the antiangiogenic treatments also altered VEGF-C and VEGF-D concentrations. Specifically, after celecoxib, both serum VEGF-C and VEGF-D levels were augmented (*p* < 0.05); meanwhile, VEGF-D levels were increased after VEGF and SU5416 inoculation, VEGF-C decreased after both treatments, and no significant differences were found after bevacizumab. With regard to tumour homogenate VEGF-C and VEGF-D concentrations, only a significant decrease in VEGF-C concentrations (*p* < 0.05) was found after VEGF and SU5416. Finally, serum IL-8 levels were significantly (*p* < 0.05) decreased after SU5416, bevacizumab and celecoxib, whereas its tumour levels were diminished (*p* < 0.05) after VEGF and SU5416 and significantly increased (*p* < 0.05) after celecoxib.

## 4. Discussion

The biological rationale behind antiangiogenic therapy use in clinical trials is based on the theory that blocking new blood vessel formation in tumours will stop or slow their growth [52]. However, the lack of substantial benefits with current antiangiogenic therapies, in terms of increased overall survival in IBC patients, remains an ongoing challenge. This study aimed to determine the effects of different antiangiogenic therapies in cell proliferation, tumour progression and tube formation in IPC-366 and SUM149 cell lines to provide new insights into the biological mechanisms involved in angiogenesis in IBC.

It has been suggested that the combination of bevacizumab and neoadjuvant regimens may have a potential benefit in patients with IBC [53]. In the present study, in vitro results showed that bevacizumab and celecoxib suppressed cell proliferation in both cell lines, as previously reported for other breast cancer cell lines [54,55]. However, after the in vivo experiments, only bevacizumab reduced tumour growth by approximately 40%, in accordance with other studies [56,57]. Therefore, our results also support the idea that bevacizumab may be a good therapeutic strategy in combination with other therapies for patients with triple negative IBC.

It is clearly established that the action of sex steroid hormones (oestrogens and androgens) is crucial in the development and progression of breast cancers [58,59], and recent evidence suggests that they might be involved in the process of angiogenesis [21]. Given the importance of sex steroids and angiogenesis in the onset, progression and metastasis of this disease, the concentrations of different steroid hormones as well as angio-genic factors were also determined to improve the knowledge of their possible role in tumour angiogenesis.

The complex microenvironment of solid tumours implies that tumour cells receive signals from multiple sources, and, conversely, they also influence the function of neighbouring cells. Apart from these paracrine signalling mechanisms, tumour cells can also acquire a certain degree of self-sufficiency by elaborating autocrine signalling pathways that facilitate key functions of growth, invasion and survival [16].

In this study, in vitro secreted P4 concentrations decreased after the addition of antiangiogenic therapies, contrary to the effect of VEGF, which did not alter the secretion of this hormone. Some authors have postulated that P4 has a role in angiogenesis through the induction of VEGF [20,21,60]. Our results are in line with these previous reports, since the inhibition of VEGF by bevacizumab decreased P4 concentrations, denoting that the cells are using P4 to induce VEGF production to survive and proliferate. Moreover, our results revealed that VEGF does no promote P4 production since no significant changes in P4 secretion were observed after the addition of VEGF to cells, supporting the idea that P4 can be a determining factor for VEGF production and cell proliferation.

On the other hand, the high secretion of T and E2 observed in the treated cells compared to the control may lead to the conclusion that these treatments do not suppress aromatase activity [61], meaning that the cells can continue to synthesise oestrogens.

Although VEGF was originally described as a pro-angiogenic factor, there is evidence of additional functions. VEGF promotes cell survival by stimulating autocrine signalling in response to extracellular stimuli [62,63,64], and also contributes to tumour migration and progression towards gradients of chemoatactants [62]. In this study, IPC-366 and SUM149 cells showed reduced levels of secreted VEGF-A after the addition of VEGF. In contrast, these VEGF-A concentrations increased significantly after the addition of bevacizumab. These results suggest that in the presence of high extracellular levels of VEGF-A, tumour cells do not secrete autocrine VEGF-A, but instead use extracellular VEGF-A to proliferate and survive.

On the other hand, the increase in E2 and VEGF-A concentrations together after treatment with bevacizumab in both cell lines could correspond to the autocrine secretion of VEGF-A by E2-induced tumour cells [65], and to an extent, this VEGF-A could act as a survival factor for tumour cells to counteract the effect of bevacizumab. Therefore, therapies with bevacizumab as a single agent do not have satisfactory results [66].

Angiogenesis is also regulated by the local activity of a variety of other angiogenic factors, such as IL-8/CXCL8, a chemokine recognised as an angiogenic factor in several cancers and promoter of tumour growth, motility and metastasis [67,68]. Moreover, IL-8 controls the expression of VEGF in endothelial cells by promoting the activation of VEGF receptors in an autocrine manner [69]. In this study, secreted IL-8 concentrations after bevacizumab were augmented, so they might exert a possible influence on the secretion of VEGF-A.

**Figure 9:**
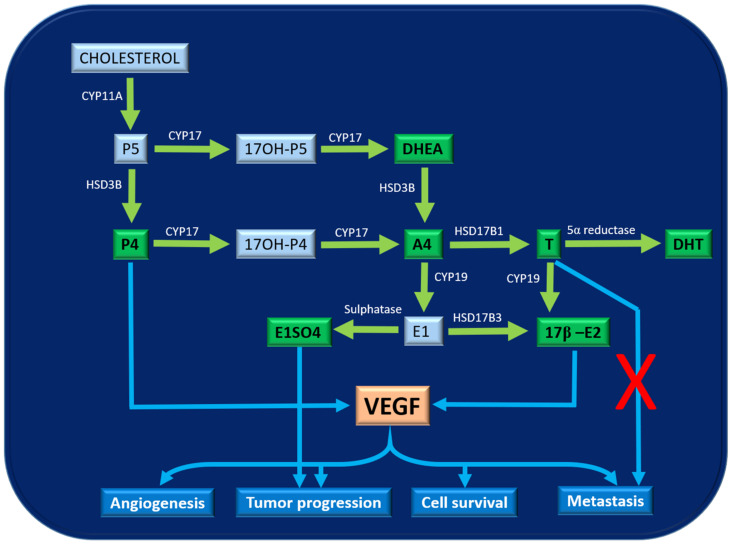
Scheme of the steroidogenic cascade. Hormones within the green box are the ones measured in this study. It can be observed that P4 and E2 induce the production of VEGF which will promote the processes of angiogenesis, tumour progression, cell survival and metastasis. Likewise, elevated E1SO4 can also promote tumour progression itself. However, an increase in T could be to be related to a lower risk of metastasis.

Therefore, our results revealed that under in vitro conditions, VEGF secretion by neoplastic cells could be regulated by P4, E2 and IL-8 concentrations. (Figure 9).

The main purpose of the use of antiangiogenic therapies is to reduce angiogenesis and thus control the tumour progression and development of metastasis since cancer growth depends on the expansion of the host vasculature. There is evidence that cancer cells can promote the formation of tubes by endothelial cells [70], but it has also been observed that cancer cells themselves are capable of forming vascular-like structures in vitro [71]. Our study has demonstrated that IPC-366 and SUM149 are capable of forming these vascular-like structures in vitro, showing their aggressiveness. Moreover, it has shown that the antiangiogenic therapies studied did not completely inhibit the formation of these structures, demonstrating that angiogenesis processes could be regulated by other factors that should be considered.

The formation of vascular structures is known to be a complex system in which the members of the VEGF family, both ligands and receptors, are involved [17]. Nevertheless, our results suggest that, apart from these proangiogenic factors, steroid hormones are also involved in tube formation, as previously observed by other authors [72]. It has been shown that the addition of androgens increases the formation of vascular-like structures [73]. Our results support this hypothesis, since the concentrations of DHEA and DHT in the culture medium decreased following the addition of VEGF to neoplastic cells, which suggests that the cells consume these androgens to increase the formation of tubular-like structures. In accordance with this, when observing the effect of bevacizumab on the number of vascular-like structures and DHT and DHEA levels, no significant differences were found, denoting that DHEA and DHT are involved in the formation of vascular-like structures.

Contrary to previously reported results [54], the results presented here clearly showed that celecoxib treatment does not inhibit the formation of vascular-like structures in vitro, and they also differ morphologically from those found in the control group. Moreover, VEGF-C concentrations were also augmented, whereas VEGF-D concentrations were reduced. VEGF-C and VEGF-D are considered lymphangiogenic factors [74]. Accordingly, celecoxib could promote lymphangiogenic processes, not only through the induction of VEGF-C but also through VEGF-D synthesis.

Moreover, after treatment with SU5416, the secreted VEGF-D concentrations were significantly decreased. SU5416 was developed as a promising selective synthetic inhibitor of the VEGFR-2/Flk-1 [75]. As VEGF-D is a ligand not only for VEGFR-2/Flk-1 but also VEGFR-3/Flk-4 [76], our data suggest that VEGF-D is possibly bound to VEGFR-3/Flk-4 as VEGFR-2/Flk-1 is inhibited by SU5416 action.

The tumour microenvironment and its hormonal secretion plays a crucial role in tumour progression. It is known that steroid hormones, such oestrogens, are involved in tumour development and progression and regulate the expression of growth factors and its receptors in breast cancer [6,21]. However, many aspects regarding the role of androgens remain unclear.

On the one hand, high urinary levels of T have been associated with worse outcomes, and an increased rate of progression has been observed in postmenopausal patients with high circulating levels of T [77]. On the other hand, there is evidence that androgens also exert an antiproliferative and apoptotic effect in breast cancer [78,79], and recently, it has been postulated that high levels of T could be associated with a lower percentage of metastasis in male mice with IMC and IBC tumours [15].

In the present study, after bevacizumab and celecoxib treatment, as intratumoural T levels were increased, oestrogen concentrations were decreased. Moreover, tumour growth rates and metastasis were reduced after both treatments in xenotransplanted mice. In the case of VEGF treatment, not only androgens but also oestrogens were significantly augmented with respect to the control group, thus promoting cell proliferation, tumour growth and metastasis. Consistent with this, our data suggest that high intratumoural T concentrations in the setting of decreased oestrogens have a role in the prevention of metastasis after both treatments.

After SU5416 treatment, E1SO4 intratumoural concentrations and tumour growth and metastasis development were augmented, although E2 concentrations were decreased. It has been postulated that E1SO4 could act as a reservoir of oestrogens for cells, and thus could promote tumour progression [80]. Moreover, some authors have associated the expression of E1SO4 with lymph node metastases and relapse free survival rate in breast cancer patients [81]. Accordingly, our data suggest that elevated E1SO4 concentrations after SU5416 treatment could promote tumour progression and the appearance of metastasis.

Although celecoxib has been reported as an antiangiogenic therapy as it inhibits prostaglandin-E2-dependent VEGF production [35], subsequently reducing microvessel density, tube formation and serum VEGF levels, several studies suggest that it may have contradictory effects on tumour responsiveness and angiogenesis [82,83]. Specifically, Ueno et al. found that VEGF levels were increased during celecoxib treatment in breast cancer patients [84]. Our findings are in line with this study, as tumour homogenate VEGF concentrations were higher after celecoxib treatment.

On the other hand, NSAIDs such as celecoxib have been reported to reduce the risk of developing cancer by suppressing the expression of inducible inflammatory cytokines, such as IL-8, by cancer cells [67]. However, the concentrations of tumour homogenate IL-8 after celecoxib were augmented in the present study. In several cancer forms, it has been demonstrated that VEGF and IL-8 are interconnected due to the upregulation of IL-8 by VEGF and vice versa [85,86]. Therefore, in view of the abovementioned findings, IL-8 could promote VEGF action, and thus, tumour progression could be encouraged.

Comparing the results of sex steroid hormone concentrations and vascular growth factors, after VEGF and bevacizumab treatments, both tumour homogenate E2 and VEGF levels appear to be positively associated, as both were significantly augmented in xenotransplanted mice treated with VEGF and significantly lower in those treated with bevacizumab. These results are in line with previous studies supporting the role of E2 as a potent regulator of VEGF in normal breast tissue [87,88] and breast cancer cell lines [89].

It has been suggested that serum hormone levels and growth factors reflect intratumoural concentrations [89]. In this study, intratumoural concentrations were higher than serum levels, which suggests that the hormones and factors are produced locally in the tissues where they act, and only a small proportion of them is released into the bloodstream.

Considering this information, the direct measurement of tumour regulators, such as VEGF and E2, locally in the tumour would be more accurate for determining the total amount of extracellular and bioactive proteins released by the tumour. Today, breast cancer is diagnosed by the histology of biopsies, mammography and gene expression levels. However, gene expression levels and intracellular protein levels are not always indicative of biological active extracellular proteins, so breast cancer patients can be over- or undertreated. Therefore, studies of the different tumour regulators and sex steroids in their bioactive compartment would substantially improve knowledge of the biological characteristics of the tumour and would provide a detailed hormone profile before treatments to predict the hormonal sensitivity of the tumour so that optimal individualised treatment could be achieved.

This study has limitations, since immunohistochemistry for angiogenesis and steroidogenic enzymes involved in these processes was not performed. While the reported changes in sex steroid and angiogenic factors provided a view of their effects on anti-angiogenic therapies, changes in the sex steroid enzymes, hormone receptors and COX-2 expression will provide a better understanding of the mechanisms involved in these treatments. Therefore, further studies are required to assess their expression.

## 5. Conclusions

This study provided evidence that steroid hormones could regulate angiogenic factors to promote tumour progression and angiogenesis. However, further studies are needed to elucidate the mechanisms by which steroid hormones could induce the production of angiogenic factors. On the other hand, considering that mammary cancer in dogs occurs spontaneously and shares clinical and pathophysiological characteristics with human cancers, this study provides new insights for further investigations for the use of these treatments as adjuvant therapy in this species. The intratumoural measurement of steroid hormones and growth factors would improve the knowledge on the biological characteristics of the tumour with the aim to develop novel preventive and individualised therapeutic strategies, as well as provide follow-up biological information regarding the effectiveness of treatments.

## Figures and Tables

**Table 1:** Evaluated hormones and antibodies used for EIA determinations.

Primary Antibody	Abbreviation	Reference
Progesterone	P4	C6E91
Androstenedione	A4	C9111
Oestrone sulphate	E1SO4	R522-2
Testosterone	T	R156
17β-oestradiol	E2	C6E91
Dehydroepiandrostenedione	DHEA	DEH3344
Dihydrotestosterone	DHT	DE2330
Vascular endothelial growth factor A	VEGF-A	RAB0107-1KT
Vascular endothelial growth factor C	VEGF-C	RAB0313-1KT
Vascular endothelial growth factor D	VEGF-D	RAB0390-1KT
Interleukin-8	IL-8/CXCL8	RAB0319-1KT

**Table 2:** Foci of pulmonary and hepatic metastasis in control and experimental groups of IPC-366 and SUM149.

		IPC-366		SUM149	
Treatments	Dosage ^a^	Foci of Pulmonary Metastasis (Mean ± SD)	Foci of Hepatic Metastasis (Mean ± SD)	Foci of Pulmonary Metastasis (Mean ± SD)	Foci of Hepatic Metastasis (Mean ± SD)
Control		13.20 ± 2.17	0.90 ± 0.60	10.40 ± 1,77	0.40 ± 0.19
VEGF	0.2 mg/Kg	13.40 ± 3.05	0.40 ± 0.15	16.40 ± 2.07	0.60 ± 0.29
	0.4 mg/kg	14.20 ± 1.92	0.60 ± 0.89	16.60 ± 2.30	2.20 ± 1.10 *
	1 mg/Kg	25.00 ± 4.85 *	3.40 ± 1.19	50.60 ± 6.95 *	4.33 ± 1.06 *
SU5416	1 mg/Kg	11.80 ± 1.48	0.40 ± 0.15	13.00 ± 5.00	1.00 ± 0.35
	5 mg/Kg	10.20 ± 3.96	0.40 ± 0.15	13.00 ± 2.83	0.40 ± 0.15
	10 mg/Kg	10.40 ± 2.30	0.20 ± 0.10	12.40 ± 2.70	0.00 ± 0.00 *
Bevacizumab	1 mg/Kg	6.80 ± 3.79 *	0.40 ± 0.15	7.40 ± 2.58	0.00 ± 0.00 *
	5 mg/Kg	1.20 ± 2.17 *	1.00 ± 0.41	0.00 ± 0.00 *	0.00 ± 0.00 *
	10 mg/Kg	0.00 ± 0.00 *	0.00 ± 0.00 *	0.00 ± 0.00 *	0.00 ± 0.00 *
Celecoxib	0.5 mg/kg	10.40 ± 2.88	0.40 ± 0.15	6.80 ± 1.82	0.00 ± 0.00 *
	2.5 mg/kg	7.00 ± 2.21	0.00 ± 0.00 *	7.40 ± 2.84	0.00 ± 0.00 *
	5 mg/kg	5.80 ± 3.10 *	0.00 ± 0.00 *	3.20 ± 1.44	0.00 ± 0.00 *

* Denoted significant differences (*p* < 0.05) between control and drug-treated groups. ^a^ Control and treated groups are composed of a total of 5 xenotransplanted mice per dosage.

## Data Availability

Data is contained within the article or supplementary material.

## Supplementary Materials

The following are available online at https://www.mdpi.com/article/10.3390/cancers13153668/s1, Table S1: Steroid (P4, DHEA, A4, T, DHT, E1SO4 and E2) concentrations, angiogenic growth factors (VEGF-A, VEGF-C, VEGF-D) and IL-8 concentrations in culture media of IPC-366 and SUM149 cell lines treated with (a) VEGF, (b) SU5416, (c) bevacizumab and (d) celecoxib, Table S2: Steroid concentrations (P4, DHEA, A4, T, DHT, SO1E4 and E2), angiogenic growth factors (VEGF-A, VEGF-C, VEGF-D) and IL-8 concentrations in tumour homogenate (H) and serum (S) in IPC-366- and SUM149 xenografted mice after (a) VEGF, (b) SU5416, (c) bevacizumab and (d) celecoxib treatments. Figure S1: In vivo effect of different dosages of VEGF, SU5416, bevacizumab and celecoxib on tumour growth in each individual IPC-366 xenotransplanted mice (*n* = 5 mice per dosage of treatment). Figure S2: In vivo effect of different dosages of VEGF, SU5416, bevacizumab and celecoxib on tumour growth in each individual SUM149 xenotransplanted mice (*n* = 5 mice per dosage of treatment).
