# Supplementary figures and images for: Anti-Angiogenic Treatments Interact with Steroid Secretion in Inflammatory Breast Cancer Triple Negative Cell Lines
Source: Cancers (Basel). 2021 Jul 21;13(15):3668. doi: 10.3390/cancers13153668 (PMC8345132; doi:10.3390/cancers13153668)

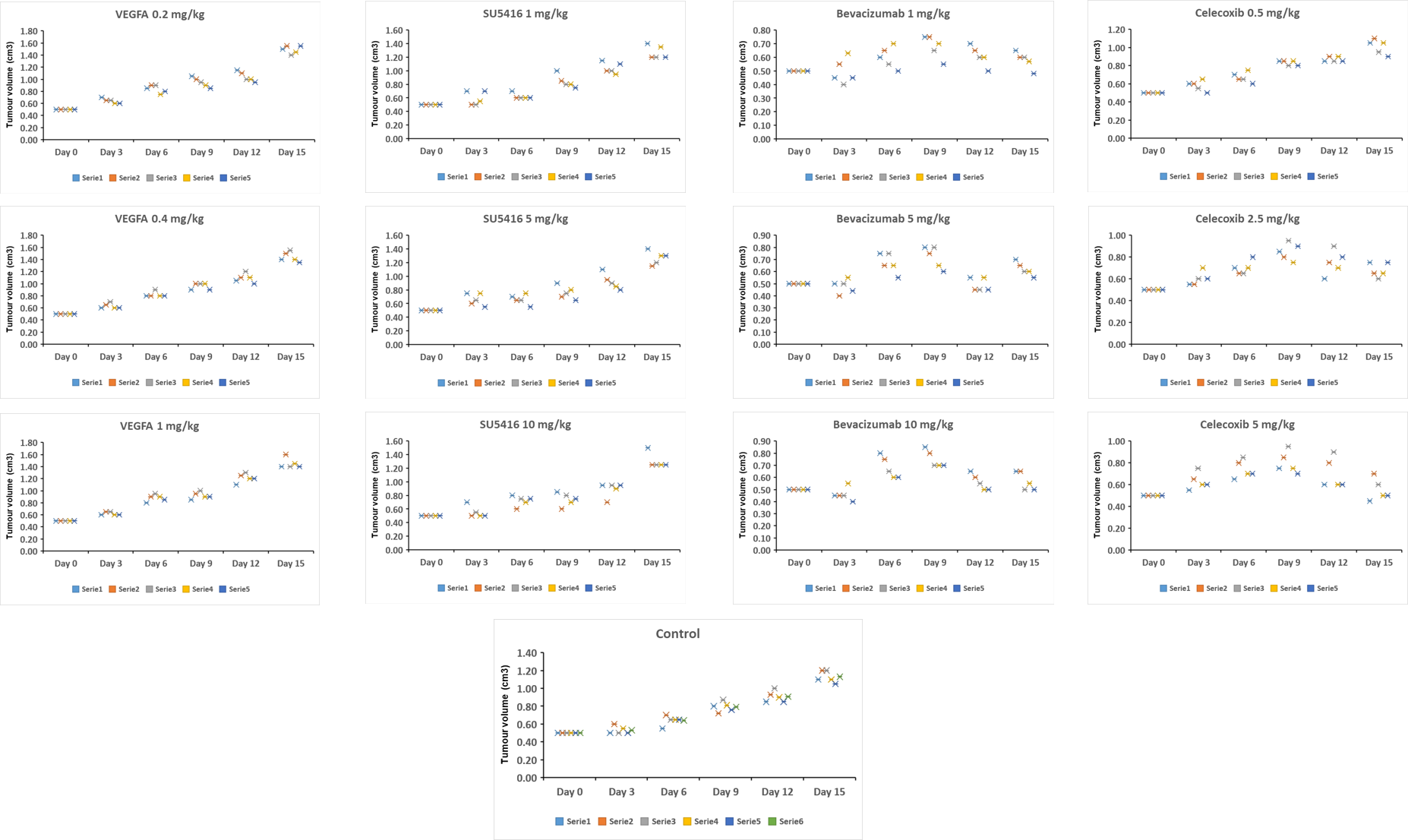

Supplement: Supplementary file 1 [file cancers-13-03668-s001.zip › FS1-IPC-366.tif]

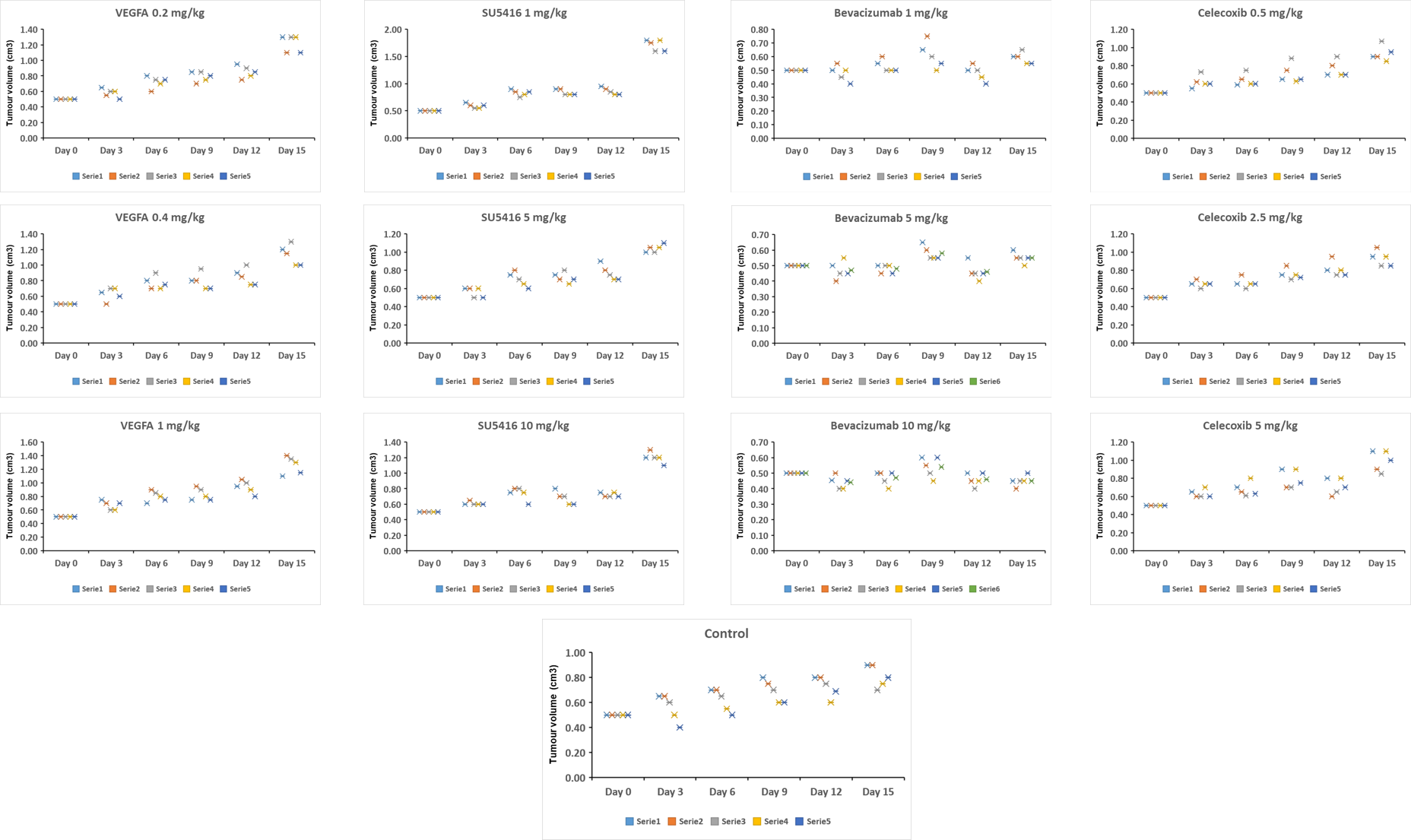

Supplement: Supplementary file 1 [file cancers-13-03668-s001.zip › FS2-SUM149.tif]
